# CD147 and Cyclooxygenase Expression in Feline Oral Squamous Cell Carcinoma

**DOI:** 10.3390/vetsci5030072

**Published:** 2018-08-13

**Authors:** Walaa Hamed Shaker Nasry, Haili Wang, Kathleen Jones, Wessel P. Dirksen, Thomas J. Rosol, Juan Carlos Rodriguez-Lecompte, Chelsea K. Martin

**Affiliations:** 1 Department of Pathology and Microbiology, Atlantic Veterinary College, University of Prince Edward Island, Charlottetown, PE C1A 4P3, Canada; wnasry@upei.ca (W.H.S.N.); hailwang@upei.ca (H.W.); jrodriguez@upei.ca (J.C.R.-L.); 2 Diagnostic Services, Atlantic Veterinary College, University of Prince Edward Island, Charlottetown, PE C1A 4P3, Canada; kjones@upei.ca; 3 Department of Veterinary Biomedical Sciences, College of Veterinary Medicine, The Ohio State University, Columbus, OH 43210, USA; dirksen.8@osu.edu; 4 Department of Biomedical Sciences, Heritage College of Osteopathic Medicine, Ohio University, Athens, OH 45701, USA; rosolt@ohio.edu

**Keywords:** feline, oral squamous cell carcinoma, CD147, EMMPRIN, Basigin, COX-1, COX-2, PGE2, inflammation, invasion

## Abstract

Feline oral squamous cell carcinoma (OSCC) is a highly invasive form of cancer in cats. In human OSCC, cluster of differentiation 147 (CD147) contributes to inflammation and tumor invasiveness. CD147 is a potential therapeutic target, but the expression of CD147 in feline OSCC has not been examined. Immunohistochemistry was used to determine if cyclooxygenase 2 (COX-2) and CD147 expression in feline OSCC biopsies was coordinated. Tumor cells were more likely to express COX-2 (22/43 cases or 51%) compared to stroma (8/43 or 19%) and adjacent oral epithelium (9/31 cases or 29%) (*p* < 0.05). CD147 was also more likely to occur in tumor cells compared to stroma and adjacent mucosa, with 21/43 (49%) of cases having >50% tumor cells with mild or moderate CD147 expression, compared to 9/28 (32%) in adjacent epithelium and only 5/43 (12%) in adjacent stroma (*p* < 0.05). In feline OSCC cell lines (SCCF1, SCCF2, and SCCF3), CD147 gene expression was more consistently expressed compared to COX-2, which was 60-fold higher in SCCF2 cells compared to SCCF1 cells (*p* < 0.05). CD147 expression did not correlate with COX-2 expression and prostaglandin E2 (PGE2) secretion, indicating that they may be independently regulated. CD147 potentially represents a novel therapeutic target for the treatment of feline OSCC and further study of CD147 is warranted.

## 1. Introduction

Oral squamous cell carcinoma (OSCC) is the most common tumor in the oral cavity of cats, accounting for 61% of oral cancers [1]. The average survival after diagnosis is only two to four months [2]. Most cats are considered to be senior at the time of diagnosis (average age of 12.5 to 13 years) [3,4]. These tumors arise most commonly from the gingiva, tongue, and sublingual region [1,4]. In the early stages, OSCC lesions often appear as small raised masses or areas of ulceration that can easily go unnoticed by the owner. By the time they are diagnosed, they tend to have already become invasive and are regularly associated with tooth loss and bone destruction when they occur near the mandible or maxilla [5]. Subsequent mortality via elective euthanasia commonly occurs when faced with this invasive disease accompanied by pain and anorexia [3].

OSCC in cats is aggressive and highly invasive, and is commonly associated with inflammation, necrosis, and ulceration [6]. The overall prognosis for cats diagnosed with OSCC is poor [3]. Treatment includes radiotherapy, chemotherapy, surgery, or combination therapy, but unfortunately, treatment efforts are usually not effective at improving survivability [5].

In some cases, feline OSCC metastasizes to regional lymph nodes and distant sites, although most cats die from local disease before the development of clinically detectable metastases [2]. Feline OSCC has important similarities with human OSCC, including its highly invasive behavior and tendency to be resistant to therapy [7]. The risk factors for feline OSCC are unknown, but flea control products, diet, and exposure to environmental tobacco smoke have been suggested as possible risk factors [8]. This is in contrast to the human form of the disease, which is highly associated with long-term tobacco and alcohol use, as well as infection with high-risk human papillomaviruses (HPV) [9,10]. The importance of HPV infection in human OSCC has inspired researchers to look for feline papillomaviruses in feline OSCC. Although the feline oral cavity can be infected with papillomavirus [11], a link between papillomavirus infection and feline OSCC has yet to be demonstrated [12].

Inflammation mediated by prostaglandin E2 (PGE2) plays an important role in the progression of a variety of human cancers, including OSCC [13,14]. Increased PGE2 production in human OSCC is attributed to increased activity of COX-2 [15]. In recent years, studies have revealed a possible role for inflammation in feline OSCC. For example, COX-2 has been shown to be expressed in a subset of feline OSCC biopsies [16]. Additionally, feline OSCC cell lines express COX-2 and STAT3 (a transcription factor important in inflammation and tumor progression), and have demonstrated sensitivity to COX inhibitors [17,18,19]. Although anti-inflammatory therapy has shown promise for the treatment of animal cancer, there are risks of gastric and renal toxicity; these drugs must be used with caution [20]. A greater understanding of the mechanisms responsible for inflammation-associated cancer progression would allow for more specific therapies to be designed, with the goal of reducing toxicity.

A surface protein called cluster of differentiation 147 (CD147, or EMMPRIN/Basigin) is known to be overexpressed in human OSCC and contributes to poor prognosis, tumor invasion, and inflammation through activation of matrix metalloproteinases (MMPs) [21,22]. MMPs are zinc-dependent endopeptidases that facilitate tumor invasion and modulate tumor-associated angiogenesis by degrading the extracellular matrix and activating a variety of mediators in the tumor microenvironment [23,24]. CD147 activation of MMPs helps tumor cells undergoing epithelial to mesenchymal transition (EMT) to invade the surrounding stroma [25]. Interestingly, Yang et al. showed that the increased expression of COX-2 in human hypopharyngeal carcinoma was associated with an increased expression of CD147 [26]. CD147-targeted therapies for human OSCC have been recently explored and demonstrated anti-tumor activity [27,28]. To date, there have not been any reports of CD147-targeted therapy for cancer in veterinary patients.

The purpose of this study was to determine if CD147 is expressed in feline OSCC biopsy tissues and cell lines, and to determine if CD147 expression is associated with COX-2 expression and PGE2 synthesis. Understanding the relationship between COX-2 and CD147 could help determine if CD147 is simply a downstream effector of PGE2 signaling in feline OSCC, or if it could be targeted alongside of COX-2 for the purpose of improved outcomes for feline OSCC patients.

## 2. Materials and Methods

### 2.1. Immunohistochemical Detection of COX Enzymes and CD147 in Feline OSCC Biopsies

#### 2.1.1. Patient Signalment and Biopsy Information

Forty-five (45) hematoxylin and eosin (HE)-stained tissue sections and corresponding paraffin-embedded tissue blocks from surgical biopsies of feline OSCC were acquired from Diagnostic Services, Atlantic Veterinary College (Charlottetown, PE, Canada). Tumors were assigned to the following anatomic locations based on information provided: tongue, sublingual (includes ventral tongue, frenulum, mouth floor), gingiva (includes tumors associated with mandible, maxilla, and jaw), soft palate, buccal mucosa, oral cavity, and undisclosed. Both genders were represented, and ages ranged from five to 17 years old (Table 1). The HE-stained tissue sections were re-evaluated microscopically by an American College of Veterinary Pathologists (ACVP) boarded veterinary pathologist to confirm the original diagnosis of OSCC.

#### 2.1.2. Primary Antibodies and Matching Isotype Control Antibodies

Immunohistochemistry protocols (IHC) were designed for the detection of cyclooxygenase-1 (COX-1), COX-2, and CD147. The COX-1 antibody was a polyclonal rabbit anti-ovine COX-1 antiserum (Cayman Chemical Company, Ann Arbor, MI, USA). The other antibodies were polyclonal rabbit anti-mouse COX-2 IgG (Cayman Chemical) and polyclonal goat anti-human CD147 IgG (Santa Cruz Biotechnology Inc., Dallas, TX, USA). Comparison of epitope sequence and predicted feline amino acid sequences available in NCBI databases revealed that the COX-2 antibody recognized an epitope that was 83% identical to feline COX-2, and the COX-1 antiserum recognized an epitope that was 91% identical to feline COX-1. The epitope sequence for the CD147 antibody was proprietary, but personal communication with the manufacturer (Santa Cruz) indicated 79% homology to feline CD147. Feline intestinal goblet cells were selected as a positive control for COX-1 [29], feline kidney (macula densa) was selected as a positive control tissue for COX-2 [30]**,** and feline small intestine was selected as a positive control for CD147 expression [31]. For negative control purposes, primary antibodies and antiserum were replaced with control rabbit IgG (Millipore Sigma Company, Etobicoke, ON, Canada), control goat IgG, and control rabbit serum (Santa Cruz).

#### 2.1.3. Immunohistochemistry Protocol

The detailed immunohistochemistry (IHC) protocol can be found in the supplementary materials. Briefly, the protocol consisted of standard horseradish peroxidase-based immunostaining of routinely deparaffinized tissue sections, and included heat-induced epitope retrieval in sodium citrate buffer (pH 6.0), the quenching of endogenous peroxidases, blocking of endogenous avidin and biotin using a commercially available kit (Vector Laboratories, Burlington, ON, Canada), 60 min of incubation with primary antibody or antiserum, and 30 min of incubation with species-appropriate biotinylated secondary antibody (Vector Laboratories). Color development was achieved with Vectastain ABC reagent (Vector Laboratories) and diaminobenzidine tetrahydrochloride (DAB) solution (Vector Laboratories). The duration of DAB incubation was determined through pilot experiments and was then held constant for all of the slides. Finally, sections were counterstained with hematoxylin, dehydrated through ethanol and xylene, and cover-slipped using a xylene-based mounting medium (Fisher Scientific, Waltham, MA, USA).

The specificity of secondary antibodies was evaluated by omitting the primary antibody from the protocol on a subset of slides. Additionally, the specificity of the protocol was evaluated by incubating positive control tissues and a subset of OSCC samples with matching control IgG and antiserum and concentrations that matched the primary antibody or antiserum. Optimization of the protocols included testing several concentrations of primary antibody, different antibody sources (CD147), and the duration of DAB incubation. A range of antibody dilutions was tested, arriving at 1:200 for COX-2 and 1:100 for COX-1 and CD147. The duration of DAB incubation ranged from 1 min to 10 min in optimization experiments before arriving at 1 min, 20 s for COX-1, 1 min, 30 s for COX-2 and 3 min, 40 s for CD147. Positive control tissues were selected based on the published literature. Feline intestinal goblet cells were selected as a positive control for COX-1 [29], feline kidney (macula densa) was selected for COX-2 expression [30]**,** and feline small intestine (enterocytes) was selected as for CD147 expression [32,33]. Positive and negative controls were included in all of the IHC runs.

#### 2.1.4. Immunohistochemistry Grading System

A visual grading system that distinguished between tumor and stroma (and adjacent epithelium if present) was developed. The percentage of positive cells in the tumor cells, adjacent epithelium, and supporting tumor stroma, along with staining intensity, was estimated and converted into a categorical grading system (Table 2).

### 2.2. Quantitative Reverse Transcriptase Polymerase Chain Reaction (RT-qPCR) for Determination of Relative Gene Expression of COX Enzymes and CD147 in Feline OSCC Cell Lines

#### 2.2.1. Cell Lines and Culture Reagents

Feline oral squamous carcinoma cells consisting of SCCF1 (laryngeal), SCCF2 (gingival), and SCCF3 (lingual) were previously developed [34,35]. OSCC cells were maintained in growth medium consisting of 90% Dulbecco’s Modified Eagle’s Medium (DMEM) (4.5 g/L glucose, HyClone Laboratories, South Logan, UT, USA) and 10% heat-inactivated fetal bovine serum (FBS, HyClone Laboratories), 100 units/mL penicillin, and 100 ug/mL streptomycin (HyClone Laboratories).

OSCC cells were grown in T75 flasks with vented caps (Fisher Scientific) and periodically split by rinsing the adherent cells with two changes of sterile PBS followed by incubating cultures in 0.25% trypsin–ethylenediaminetetraacetic acid (trypsin-EDTA) (Wisent Inc., Saint-Jean-Baptiste, QC, Canada) until cells lifted from the surface of the flask (5 min to 10 min). Trypsin was neutralized by the addition of serum-containing growth medium, and a fraction of the cells (1/10 to 1/20) were moved to a new flask with fresh medium.

#### 2.2.2. Experimental Conditions, RNA Extraction, and cDNA Synthesis

Cells were trypsinized and counted using a hemocytometer and trypan blue exclusion, and 2 × 10^5^ cells were seeded into each well of a six-well plate (Fisher Scientific). Cells were grown for 48 h, rinsed with sterile PBS, and their regular medium was replaced with serum-free medium containing 0.1% bovine serum albumin (BSA) (HyClone Laboratories) and cultured overnight. The next morning, the serum deprivation group remained in the serum-free medium for an additional 2 h, while the medium for the serum-stimulated group was replaced with standard growth medium containing 10% FBS and cultured for 2 h. This protocol provided cultures that were approximately 80% confluent at the time of ribonucleic acid (RNA) extraction. Cells were cultured in triplicate wells, and each experiment was repeated for a total of three independent experiments.

RNA was extracted using the Aurum total RNA mini kit (Bio-Rad Laboratories, Mississauga, ON, Canada) according to the manufacturer instructions, which included DNase digestion in order to reduce genomic DNA contamination. RNA was eluted in 40 μL of elution buffer. RNA concentration and A260/A280 ratios were determined using the NanoDrop ND–1000 spectrophotometer (Thermo Scientific, Rockford, IL, USA). Isolated RNA was stored at −80 °C until used for RT-qPCR.

RNA samples were reverse transcribed using the iScript gDNA Clear cDNA Synthesis Kit (Bio-Rad Laboratories) following the manufacturer instructions (250 ng of RNA in a 20-μL reaction volume). In order to test for genomic DNA contamination, no reverse transcriptase (NRT) controls were prepared on a subset of samples. A Bio-Rad thermal My-Cycler was used for the synthesis reaction (5 min at 25 °C, reverse transcription for 20 min at 46 °C, and RT inactivation for 1 min at 95 °C). cDNA was stored at −20 °C until used for real-time PCR.

#### 2.2.3. Primer Design and RT-qPCR Optimization 

Primers were designed using feline gene sequences catalogued in the databases of the National Center for Biotechnology Information (NCBI, Bethesda, MD, USA), and Primer-Blast software (NCBI). Candidate primer pairs were prioritized based on melting temperatures (Tm) near 60 °C and within one degree of each other, the presence and length of a spanned intron, and the size of the amplicon (Table 3). Three primer pairs were initially designed for each target, and the primer pair that had the best amplification and qRT-PCR product melting characteristics for each target was chosen. Primer validation included checking for the appropriate size of the PCR product on a 1% agarose gel, and sending PCR products and primers to an outside laboratory to determine the sequence (ACGT Corporation, Toronto, ON, Canada). Product sequences were compared to feline complementary deoxyribonucleic acid (cDNA) sequences using NCBI Primer Blast software in order to determine if the primers amplified the desired target. Annealing temperatures were optimized using thermal gradients and pooled cDNA samples, and standard curves were evaluated to determine that reaction efficiencies were between 90% and 110% (additional detail in the supplemental materials). Appropriate performance in no-RT (NRT) and no-template control (NTC) reactions were confirmed. For NTC reactions, cDNA was replaced with nuclease-free water. Real-time PCR was performed using a CFX96 Real-Time PCR Machine (Bio-Rad Laboratories). See the supplementary materials for specific PCR details, including reaction components, cDNA dilution factors, and reaction protocol. Gene stability values for the reference genes were revaluated in the final data sets using CFX Manager Software 3.1 (Bio-Rad Laboratories, Mississauga, ON, Canada). The reference genes selected for normalization in the final analysis were HPRT1, RPS18, and B2M.

### 2.3. Enzyme-Linked Immunosorbent Assay (ELISA) Detection of PGE2 in Feline OSCC Cell Lines

To measure PGE2 secretion, feline OSCC cells were seeded into six-well plates at a density of 5 × 10^5^ cells per well. OSCC cells were cultured in growth medium as described above for 48 h. Supernatants were collected at 48 h, and adherent cells were counted using a hemocytometer after trypsinization. The experiment was performed in triplicate wells, and repeated for a total of three independent experiments. PGE2 levels in the supernatants were measured using a Prostaglandin E2 Multispecies Competitive ELISA Kit (Thermo Scientific), according to the manufacturer instructions. PGE2 concentration was calculated from a standard curve and normalized to the average number of cells in the cultures at the time of supernatant collection in order to accommodate for different rates of cellular proliferation of the three cell lines over the course of the experiment. PGE2 concentration is expressed as an x-fold increase over SCCF1 cells.

### 2.4. Statistical Analysis

All of the statistical analyses were carried out using Stata IC 14 software (StataCorp LLP, College Station, TX, USA). P values equal to or less than 0.05 were considered statistically significant. Fisher’s exact test was used for categorical immunohistochemistry data. In vitro experiments were performed with triplicate cultures, with each sample analyzed in duplicate, and each experiment was repeated for a total of three independent experiments. Data from the three experiments were combined for statistical analysis. RT-qPCR and ELISA data was tested for normality using the Shapiro–Wilk test, followed by the Kruskal–Wallis test for non-parametric data. It is graphically represented by showing relative expression compared to a low-expressing cell line. PGE2 ELISA data was normalized to the average cell number, and expressed as the fold change relative to the PGE2 concentration from SCCF1 cells. Bar graphs represent means with standard deviation.

## 3. Results

### 3.1. Immunohistochemical Detection of COX Enzymes and CD147 in Feline OSCC Biopsies

**Figure 1:**
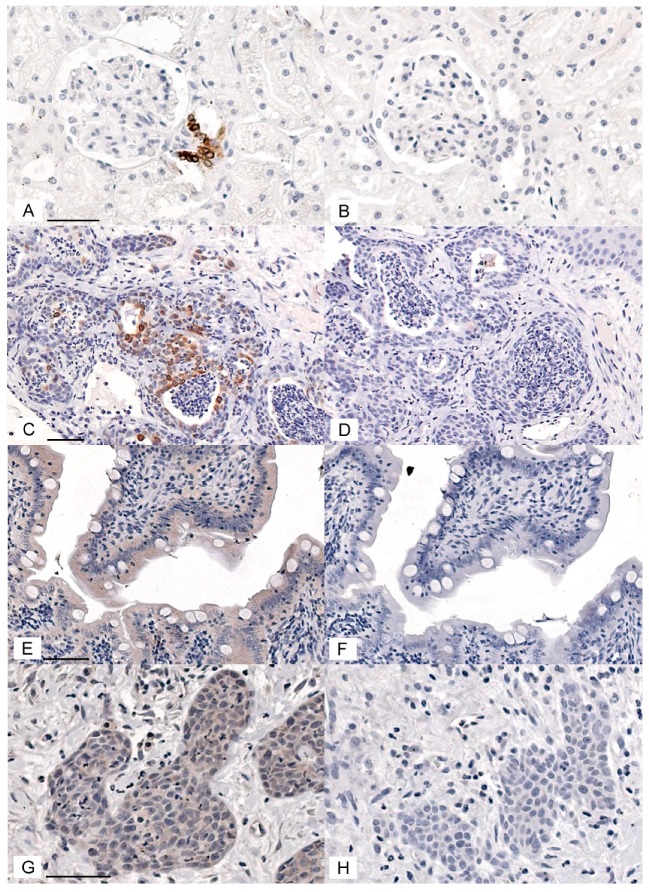
COX-2 and CD147 positive and negative controls for immunohistochemistry. (**A**–**D**) Photomicrographs of IHC staining using rabbit anti-COX-2 IgG (1:200) and normal rabbit IgG (1:200, negative control). The chromogen is DAB (brown), and the counterstain is hematoxylin (blue). The feline renal macula densa epithelial cells had an intense cytoplasmic signal (**A**) that was eliminated when the antibody was replaced with normal IgG (**B**). Scattered oral squamous cell carcinoma (OSCC) cells showed an intense cytoplasmic COX-2 signal (**C**), which was eliminated when the primary antibody was replaced with normal IgG (**D**). (**E**,**F**) IHC staining using goat anti-CD147 IgG (1:100) and normal goat IgG (1:100, negative control). Feline enterocytes showed moderately intense cytoplasmic and membranous signal (**E**), which was markedly reduced when the primary antibody was replaced with normal goat IgG (**F**). There was widespread moderate staining and scattered heavy staining of OSCC cells (cytoplasm and membrane), and light to moderate widespread staining of stromal cells (**G**). Staining was markedly reduced when the primary antibody was replaced with normal goat IgG (**H**). Each pair of positive and negative control images are at the same magnification. Scale bars are 50 μM long.

In order to evaluate COX-1, COX-2, and CD147 expression in feline OSCC, immunohistochemical protocols were developed and optimized. COX-2 immunohistochemistry gave positive signal in feline macula densa cells and in feline OSCC (Figure 1A,C). Replacing the primary antibody with control rabbit IgG eliminated the staining (Figure 1B,D), CD147 immunohistochemistry gave a positive signal in feline small intestine enterocytes, as well as feline OSCC (Figure 1E,G). Replacing the CD147 antibody with control goat IgG significantly reduced staining (Figure 1F,H). Rabbit anti-COX-1 antiserum yielded a positive signal in feline intestinal goblet cells and feline OSCC (Figure S1). Unfortunately, replacing the COX-1 antiserum with normal rabbit serum (negative control) demonstrated reduced staining in goblet cells but a high amount of nonspecific stain that was indistinguishable from the COX-1 anti-serum in feline OSCC (Figure S1). COX-1 immunohistochemistry was not selected for further analysis.

**Figure 2:**
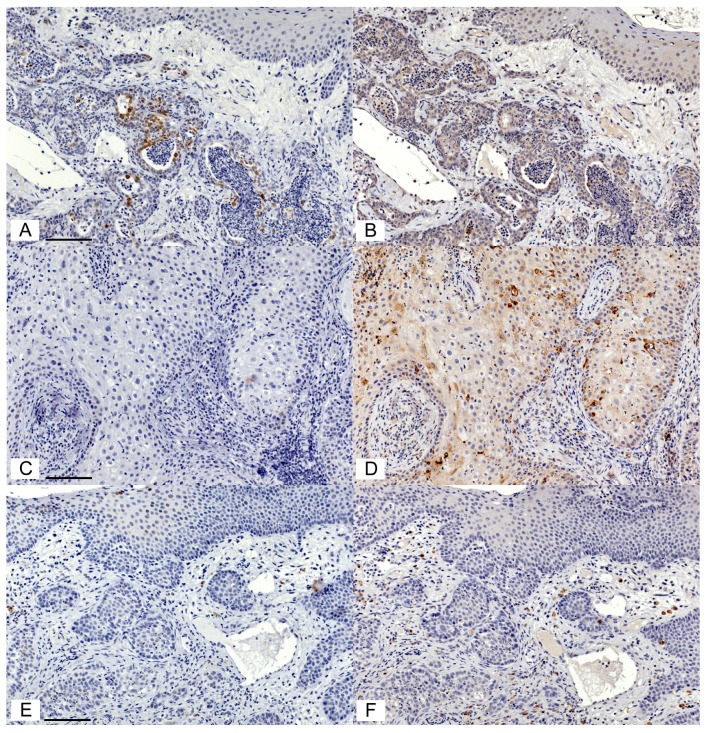
COX-2 and CD147 expression varied between cases of feline OSCC. Photomicrographs of IHC staining using rabbit anti-COX-2 IgG (1:200) and goat anti-CD147 IgG (1:100). The chromogen is DAB (brown), and the counterstain is hematoxylin (blue). Case 1: There are scattered OSCC cells with an intense COX-2 signal (**A**). In contrast, OSCC cells showed widespread light to moderate CD147 signal (**B**). Case 2: This sample was COX-2 negative (**C**), but showed widespread moderate staining and scattered heavy staining for CD147 (**D**). Case 3: This sample was COX-2 negative (**E**) and CD147 negative in tumour cells (**F**), but had scattered moderate CD147 staining in the stroma (**F**). All images are at the same magnification. Scale bars are 100 μM long.

**Figure 3:**
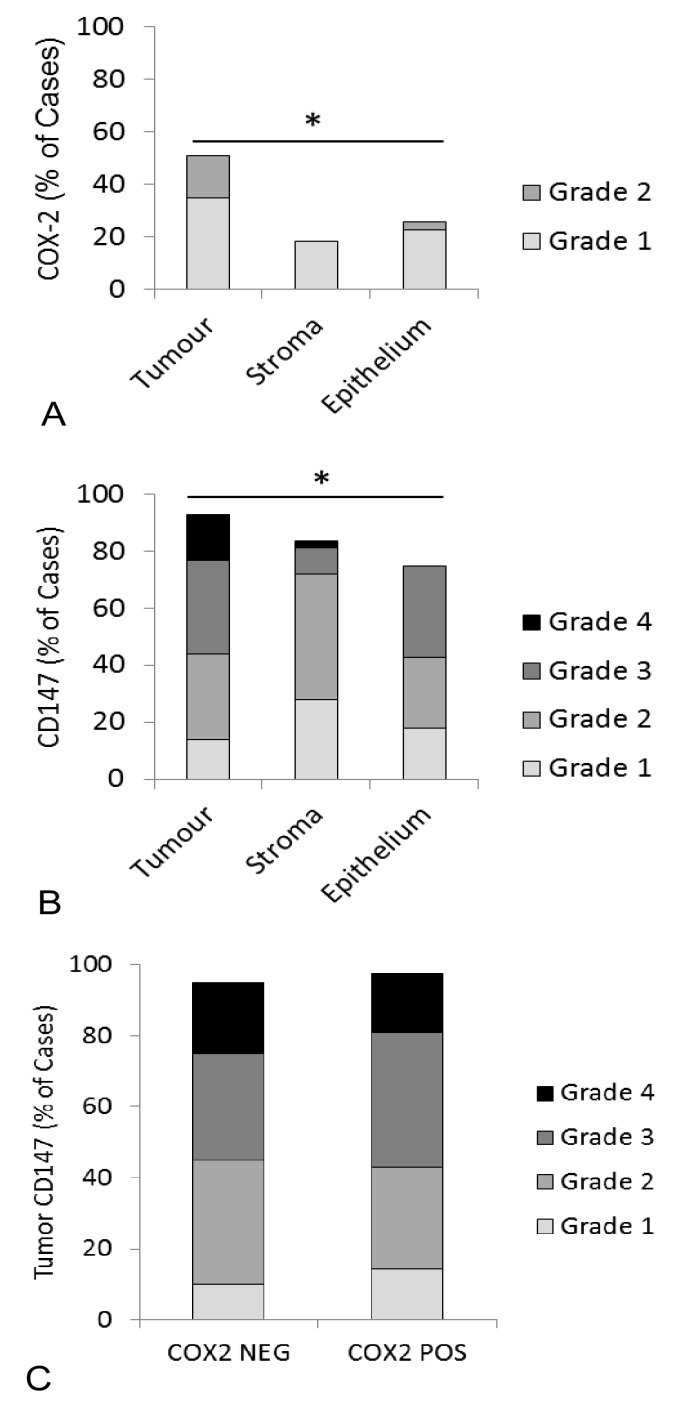
Expression of COX-2 and CD147 in feline OSCC samples using an IHC grading system. (**A**) COX-2 staining results were obtained from 43 samples, 31 of which included adjacent oral epithelium. Each bar represents the percentage of cases that were considered positive for COX-2 expression within each compartment (tumor cells, stroma, and adjacent epithelium). Each bar is subdivided to demonstrate the percentage of cases assigned to each grade. COX-2 expression was highest in the tumor cells. (**B**) CD147 staining results were obtained from 43 samples, 28 of which included adjacent oral epithelium. CD147 expression was highest in the tumor cells. (**C**) Paired COX-2 and CD147 IHC results were available from 42 OSCC cases. Twenty-two (22) cases had positive COX-2 expression in the tumor cells (grades 1 and 2) and 20 cases were COX-2 negative in the tumor cells (grade 0). Each bar represents the percentage of positive CD147 cases within the COX-2 positive and COX-2 negative groups. There was no significant difference in CD147 expression between COX-2 positive and COX-2 negative cases. There were no cases with grade 5 staining. All of the statistical comparisons were made using Fisher’s exact test (* *p* value < 0.05).

Serial sections of feline OSCC were stained for COX-2 and CD147 expression yielding variable levels of positive signal (Figure 2). Of the 45 feline OSCC samples, two were removed from COX-2 IHC and two were removed from CD147 IHC due to the drying of slides during processing. Using a visual grading system, the percentage of positive cells in tumor, stroma, and overlying epithelium (lacking morphologic evidence of malignancy) was estimated and converted into categorical data. Although COX-2 expression was generally low, COX-2 was more likely to be expressed in tumor cells compared to the surrounding stroma or adjacent epithelium. Fifteen of 43 samples (34%) had 1–9% positive tumor cells (grade 1), and 7/43 (16%) had 10–50% positive tumor cells (light intensity, grade 2), in contrast with adjacent epithelium, which was only positive in 8/31 (23%, grade 1) and 1/31 (3%, grade 2). Similarly, stroma was only COX-2 positive in 8/43 cases (19%, grade 1) (*p* < 0.05, Figure 3A). CD147 was also more likely to occur in tumor cells compared to stroma and adjacent mucosa. 21/43 (49%) OSCC had >50%, mild, or moderate CD147-positive tumor cells (grade 3 and 4), compared to 9/28 (32%) OSCC with grade 3 or grade 4 staining in adjacent epithelium and only 5/43 (12%) of OSCC cases with that level of staining in the surrounding stroma (*p* < 0.05, Figure 3B). CD147 expression in the tumor compartment was similar between COX-2 positive (grade 1 and 2) and COX-2 negative tumors. More specifically, 11/21 (52%) of COX-2 positive tumors had grade 3 or 4 CD147 expression, compared to 10/20 (50%) of COX-2 negative tumors (Figure 3C).

### 3.2. Expression of COX-1, COX-2, and CD147, and Secretion of PGE2 in Feline OSCC Cell Lines

**Figure 4:**
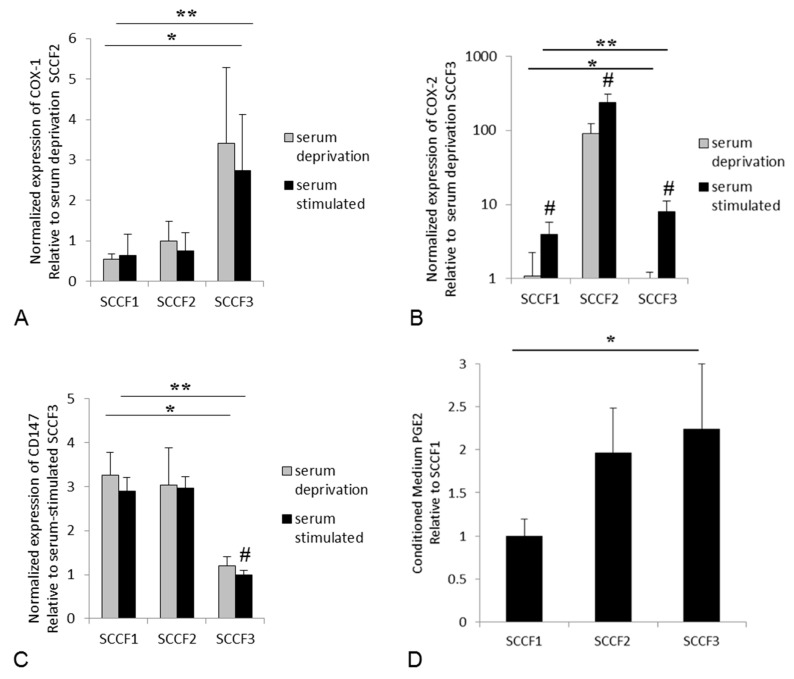
COX-1, COX-2, and CD147 mRNA expression, and prostaglandin E2 (PGE2) secretion from feline OSCC cell lines in vitro. (**A**) Each bar represents the mean fold increase in COX-1 mRNA compared to serum-deprived SCCF2 cells. There was no significant effect of serum exposure on COX-1 expression. The expression of COX-1 differed between the three cell lines in serum deprived (*) and stimulated (**) conditions, with SCCF3 expressing the most COX-1, and SCCF1 cells expressing the least COX-1. (**B**) Each bar represents the mean fold increase in COX-2 mRNA compared to serum-deprived SCCF3 cells. Serum exposure significantly stimulated COX-2 expression in all three cell lines. The expression of COX-2 differed between the three cell lines in serum-deprived (*) and stimulated (**) conditions, with SCCF2 cells expressing the most, and SCCF1 cells expressing the least. (**C**) Each bar represents the mean fold increase in CD147 mRNA compared to serum-stimulated SCCF3 cells. Serum exposure only had a significant effect on the SCCF3 expression of CD147. The expression of CD147 differed between the three cell lines in serum-deprived (*) and stimulated (**) conditions, with SCCF3 cells expressing the least CD147. (**D**) Each bar represents the mean PGE2 concentration relative to conditioned medium from SCCF1 cells. SCCF1 cells secreted the least PGE2. The average concentration of PGE2 in SCCF1-conditioned medium was 22.2 pg/mL per 100,000 cells. Each graph represents combined data from three independent experiments. All of the statistical comparisons of RT-qPCR data were made using the Kruskal–Wallis test (* serum-deprived, *p* value < 0.05; ** serum-stimulated, *p* value < 0.05; # serum-deprived compared to serum-stimulated, *p* value < 0.05). PGE2 concentrations were compared using the Kruskal–Wallis test (* *p* value < 0.05).

COX-1, COX-2, and CD147 expression was detected at the mRNA level in all of the feline OSCC cell lines. Serum-stimulation up-regulated COX-2 in all of the cell lines, but had no significant effect on COX-1 expression, and only significantly affected CD147 expression in the feline SCCF3 cells (Figure 4). The expression of all three genes significantly differed between cell lines, regardless of whether the cells were serum-deprived or serum-stimulated cells (Figure 4). SCCF1 cells expressed the least COX-1 and COX-2, SCCF2 cells expressed the most COX-2, and SCCF3 cells expressed the most COX-1. This expression pattern did not appear to be related to CD147 expression, since CD147 was expressed similarly between SCCF1 and SCCF2 cells, with SCCF3 expressing the least CD147. SCCF1 expressed relatively low levels of COX-1 and COX-2, which corresponded with this cell line secreting the least PGE2 compared to SCCF2 and SCCF3 cells (Figure 4).

## 4. Discussion

This study represents the first time that CD147 has been investigated in cats. CD147 expression in feline OSCC was variable; however, it was generally widespread throughout the tumor cells, stromal compartment, and adjacent gingiva, and was expressed in all three feline OSCC cell lines. Importantly, CD147 expression was highest in the tumor cells compared to stroma and the adjacent mucosal epithelium. Although there is no veterinary literature specific to CD147 that can be referred to, it appears that CD147 is expressed in feline OSCC similar to what is reported for human OSCC. Huang et al. observed that in people, CD147 expression was increased in lingual OSCC compared to adjacent tissues [22], and Vigneswaran et al. demonstrated that CD147 expression increased in premalignant lesions and OSCC compared to normal oral mucosal cells [36]. This concept has been echoed in a more recent study by Ma et al., who revealed that CD147 mRNA was increased in human OSCC tumor tissue compared to normal adjacent tissues, and was higher in a human OSCC cell line (Tu686) compared to an immortalized non-tumorigenic cell line (HaCaT). Furthermore, they found that CD147 expression was associated with multidrug resistance, and they concluded that CD147 plays a crucial role in human OSCC progression [37].

Since CD147 is known as an inducer of MMP activity, we can infer from MMP-related studies in the veterinary literature that CD147 might possess similar functions in animals. A recent review article describes that MMPs are present in a variety of animals, including cats [38]. For example, studies have shown that MMPs are expressed in feline tumors, including MMP9 and MMP2, in feline meningioma and mammary cancer [39,40], and MMP9 in feline lymphoma [41]. Therefore, it is not surprising that the CD147, as an inducer of MMP activity, would have a role in feline tumors as it does for human cancer.

In this study, feline OSCC expression of CD147 was similar to COX-2 in that higher expression was more likely to occur in the tumor cells rather than the stroma and adjacent epithelium. Increased COX-2 expression in feline OSCC was also reported by Hayes et al., who found that COX-2 staining was higher in tumor cells (67%) compared to adjacent histologically normal oral mucosa, which was negative [16]. While there were more COX-2 positive cases in the Hayes study than our current study, it is important to note that the majority of their positive cases had less than 1% positive tumor cells, which was considered ”negative” in our grading scheme. A similar study by DiBernardi et al. showed that six out of 34 (~18%) feline OSCC cases had high expression of COX-2, 22 (~64%) cases had weak expression, and six (~18%) cases had none [2]. These findings were in contrast with Beam et al., who found that COX-2 was only expressed in two out of 21 (~9%) feline OSCC biopsies. They also found that COX-2 had low expression in feline transitional cell carcinomas, and had no expression in cutaneous squamous cell carcinomas, adenocarcinomas (mammary, pulmonary, and intestinal), lymphomas, or vaccine-associated sarcomas [42]. On the other hand, others have reported that feline mammary cancer does express COX-2 [43], which illustrates the wide variability in IHC staining results across the published literature.

COX enzymes contribute to a cycle of chronic inflammation through the production of PGE2, although other elements in the arachidonic acid cascade are important for overall PGE2 levels. ELISA was performed in order to determine if COX-2 expression was indeed correlated with PGE2 synthesis. Interestingly, the sharp increase in COX-2 expression from SCCF2 cells did not translate into pronounced PGE2 secretion compared to the other cells. In fact, SCCF3 tended to have higher PGE2 secretion, and it was also the cell line with the highest COX-1 expression. These findings suggest that OSCC expression of COX-1 could be contributing to PGE2 level and tumor-associated inflammation, despite most of the attention being paid to COX-2 expression (and COX-2 inhibitors) in the OSCC-related literature. Other factors regulating PGE2 levels include PGE2 synthase enzymes downstream of COX enzymes that convert PGH2 into PGE2, and 15-hydroxyprostaglandin dehydrogenase (PGDH), which is an enzyme that inactivates PGE2 [44]. Further study is needed in order to determine if these other players impact levels of PGE2 in OSCC.

The importance of the COX pathway of inflammation in other animal diseases has been reported. For example, in dogs, COX-2 expression has been demonstrated in OSCC [45], and may serve as a prognostic indicator in malignant mammary tumors [46]. Additionally, piroxicam combined with etoposide induced synergistic anti-proliferative effects on canine osteosarcoma [47]. PGE2 has been detected in human and canine prostate cancer [48], and microsomal PGE synthase-1 (mPGES-1) and EP2 receptor have been shown to be expressed in canine, feline, and human mammary cancer [46]. COX-2, COX-1, and PGE2 have been demonstrated in the feline intestinal tract as they have in people, with cats being susceptible to intestinal lesions secondary to NSAID usage, suggesting that the COX pathway functions similarly in the two species [29,49]. In contrast, a study looking at COX-2 expression in feline pancreatic adenocarcinoma revealed that COX-2 was not consistently expressed like it is in human pancreatic cancer, and that COX-2 inhibitors for the treatment of the feline form of the disease may not be a good choice [50]. This indicates that despite the similarities, it cannot be assumed that the COX pathway is equally important in feline and human tumors. This highlights the importance of studying animal cancer using species-specific samples and cell lines, rather than relying on extrapolation from the human OSCC literature.

It is important to note that CD147 and COX expression, as well as PGE2 secretion, varied across the feline OSCC cell lines, corroborating the varying expression of COX-2 and CD147 observed in feline OSCC tumor tissue. This emphasizes why multiple cell lines should be included in in vitro studies whenever possible, in order to try and capture the heterogeneity of feline OSCC patients.

In this study, COX-2 and CD147 were increased in tumor cells compared to stroma and adjacent oral mucosa, supporting the concept that each of these factors could represent therapeutic targets. Interestingly, CD147 expression did not appear to be correlated with COX expression and PGE2 secretion, suggesting that the expressions of CD147 and COX-2 are separately regulated, and they each could have their own role to play in feline OSCC progression. For example, our previous work showed that the high-COX-2 expressing SCCF2 cells were more bone-invasive in an orthotopic nude mouse model of feline OSCC compared to SCCF1 and SCCF3, and the SCCF2 cells expressed the most parathyroid hormone related-protein (PTHrP), a known mediator of tumor invasion into bone [35]. In contrast, SCCF1 cells were minimally invasive in nude mice and expressed the least PTHrP of these three cell lines [35]. They also expressed relatively low COX-2 and secreted the least PGE2. PTHrP expression has been shown to be stimulated by PGE2 in synovial cells in rheumatoid arthritis [51], and perhaps a similar relationship is true in feline OSCC cells. CD147 expression in SCCF1 and SCCF2 cells were similar, suggesting that the mere presence of CD147 is not sufficient for feline OSCC invasive ability. Since CD147 exerts its effects on invasiveness via the activation of MMPs, inadequate MMP expression, or increased tissue inhibitor of matrix metalloproteinases (TIMPs) [52], could prevent invasiveness despite the expression of CD147. In other words, CD147 may be required for the invasive behavior of feline OSCC, but it is not sufficient on its own. Future studies are needed in order to determine the role of CD147 in feline OSCC invasiveness.

## 5. Conclusions

For the first time, CD147 has been shown to be expressed in feline OSCC, and is a candidate target for feline OSCC treatment that may be independent of PGE2-related inflammation. Future studies will evaluate the role of the CD147 in feline OSCC in terms of MMP activity and tumor cell invasiveness. Additionally, cyclooxygenase activity remains as a potential therapeutic target for feline OSCC, and deserves continued investigation.

## Figures and Tables

**Table 1:** Feline oral squamous cell carcinoma (OSCC) cases. DSH: domestic short hair, DMH: domestic medium hair, DLH: domestic long hair.

Gender	(N)	Breed	(N)	Age (Years)	(N)	Tumor Location	(N)
Male	24	DSH	16	5–7	3	Gingiva	8
Female	19	DMH	2	8–10	10	Sublingual	14
		DLH	9	11–13	16	Tongue	5
		Persian	1	14–17	15	Buccal Mucosa	5
		Burmese	1			Palate	1
						Oral cavity	7
Undisclosed	2	Undisclosed	16	Undisclosed	1	Undisclosed	5

**Table 2:** Immunohistochemistry grading system.

Grade	Descriptions
Negative	less than 1% positive cells
Grade 1	1–9% positive cells (light, moderate or heavy staining)
Grade 2	10–50% light cells with scattered moderate or heavy staining
Grade 3	mostly light, <50% moderate with scattered heavy staining
Grade 4Grade 5	mostly moderate, <50% heavy staining>50% heavy staining

**Table 3:** RT-qPCR primers * Genes of interest: PTGS1 (COX-1), PTGS2 (COX-2), and CD147. ** Reference genes that were selected for normalization of the data after evaluation of M value (GeNorm M value) and CV value (coefficient of variation). Unmarked genes are candidate reference (housekeeping) genes that were tested but not selected.

Primer Name	Primer Sequence	Amplicon Length	Intron-Spanning
* fPTGS1-S	GTCCTTCAACAGGGACTGGAC	129	no
fPTGS1-4-AS	ATGCTGGTTACTTATCTCGCTCC		
* fPTGS2-S	GGCGTGAACCACGAGAAGTA	136	yes
fPTGS2-AS	GATGGCATGGACTGTGGTCA		
* fCD147-S	ATTGACCCCACCGGTACCTA	95	yes
fCD147-AS	CCCAAAAGGACCTGAGCGAA		
fTUBB-S	CAAGCGGTTTGCTGCTGTTA	159	yes
fTUBB-AS	GCTAGTCGGGACTGCTCTTC		
fTBP-S	TCTATGAGAAGCGACGGAAGC	117	yes
fTBP-AS	GCCTTTGTCGTTGATGTGCC		
** fRPS18-S	TTGCCCAACACTTCACCCAT	92	yes
** fRPS18-AS	AGGCGCAGTTTATGCTGTCT		
** fHPRT1-S	AATTGGGGCCCCCTTTTCTC	158	no
** fHPRT1-AS	CCACCAAATGTGCTTGGCTT		
fGAPDH-S	TGTCCATCCTTCGTCCCTCA	144	yes
fGAPDH-AS	CGGACACTCCAGAACCGTAG		
** fB2M-S	GTTCTCTCTTCCACAGGAGGC	146	yes
** fB2M-AS	ACTCGCAAAATGTGCTGGAAC		

## Supplementary Materials

The following are available online at http://www.mdpi.com/2306-7381/5/3/72/s1, Supplemental Methods: Immunohistochemistry protocol, RT-qPCR protocol, RT-qPCR standard curves and reaction efficiency; Figure S1: COX-1 positive and negative controls for immunohistochemistry.
