# Supplementary material for: CD147 and Cyclooxygenase Expression in Feline Oral Squamous Cell Carcinoma
Source: Vet Sci. 2018 Aug 13;5(3):72. doi: 10.3390/vetsci5030072 (PMC6163611; doi:10.3390/vetsci5030072)
Supplement: Supplementary file 1 [file vetsci-05-00072-s001.pdf]

Supplemental Materials

Methods

Immunohistochemistry protocol

Immunostaining was performed on paraffin-embedded archival tissue. In brief, the paraffin blocks were sliced into 5-µM thick sections, deparaffinized with xylene (Fisher Scientific, Waltham, MA, USA), and rehydrated with decreasing concentrations of ethanol in water. Antigen retrieval was achieved by preheating sodium citrate buffer (pH 6.0) for two minutes in a 750-W microwave oven, followed by incubation of the slides for 20 min in the hot (95 ^{0}C) citrate buffer in a steamer (Black and Decker, Mississauga, ON, Canada), and 20 min of cooling at room temperature.

Endogenous peroxidases were quenched by incubating the slides in 3% hydrogen peroxide (Fisher Scientific) for 20 min. The sections were then washed with phosphate-buffered saline (PBS) (Fisher Scientific) for 10 min. Endogenous avidin and biotin was blocked using a blocking kit according to manufacturer instructions (Vector Laboratories, Burlington, ON, Canada). The sections were then washed with PBS for four minutes. Primary antibodies were applied for 60 min at room temperature in a humidified chamber. After rinsing the slides in PBS, they were incubated in secondary antibody (1:100 biotinylated goat anti-rabbit IgG or rabbit anti-goat IgG (Vector Laboratories) for 30 min at room temperature. After washing with PBS for 10 minutes, the slides were incubated with Vectastain ABC reagent (Vector Laboratories) for 30 min. After washing with PBS for five minutes, color development was achieved by applying diaminobenzidine tetrahydrochloride (DAB) solution (Vector Laboratories) for two to five minutes, depending on the primary antibody. The duration of DAB incubation was determined through pilot experiments and was then held constant for all of the slides. After washing in distilled water, the sections were counterstained with haematoxylin (Vector Laboratories), dehydrated through ethanol and xylene, and cover-slipped using a xylene-based mounting medium (Fisher Scientific).

RT-qPCR protocol

The PCR reaction included 0.5-µM forward primer and 0.5-µM reverse primer (1 µL combined volume), 5 µL of SYBR Green Supermix (Biorad), and 4 µL of diluted cDNA (or NRT or water). Finalized RT-qPCR protocols used cDNA diluted 1:16 (COX-1, COX-2, CD147, TBP and HPRT1) and 1:64 (B2M, GAPDH, TUBB and RPS18). All of the samples in each experiment were run in technical duplicates (two reaction wells per sample generating an average Cq value). Reaction protocols started with a three-minute 95 °C denaturation step followed by 40 cycles consisting of five seconds of denaturation at 95 °C, 20 s of annealing at 60 °C (except for RPS18 and HPRT1 which were at 63 °C), and 20 s of extension at 72 °C. The reaction protocol was completed with a melt curve analysis.

RT-qPCR standard curves and reaction efficiency

In order to determine the reaction efficiencies of the primer pairs, serial dilutions of pooled cDNA were prepared from the cell line samples. The dilution series ranged from twofold dilution series to fourfold dilution series, depending on the Cq values generated during the thermal gradient experiment. Negative controls (NTC) were included with all standard curves. The efficiency of the optimized RT-qPCR assays was determined using CXF manager software (Bio-Rad CFX Manager 3.1). Target amplification efficiency for the reactions ranged from 90–110%. The feline OSCC cells demonstrated very low COX-1 expression, which made calculating the reaction efficiency difficult (data from a minimum of five dilutions was required to calculate the efficiency). Reaction efficiency for the feline COX-1 primers was determined using cDNA synthesized from frozen feline kidney samples collected from a recently euthanized cat from the Prince Edward Island (PEI) Humane Society (approved by the University of Prince Edward Island Animal Care Committee).

Results

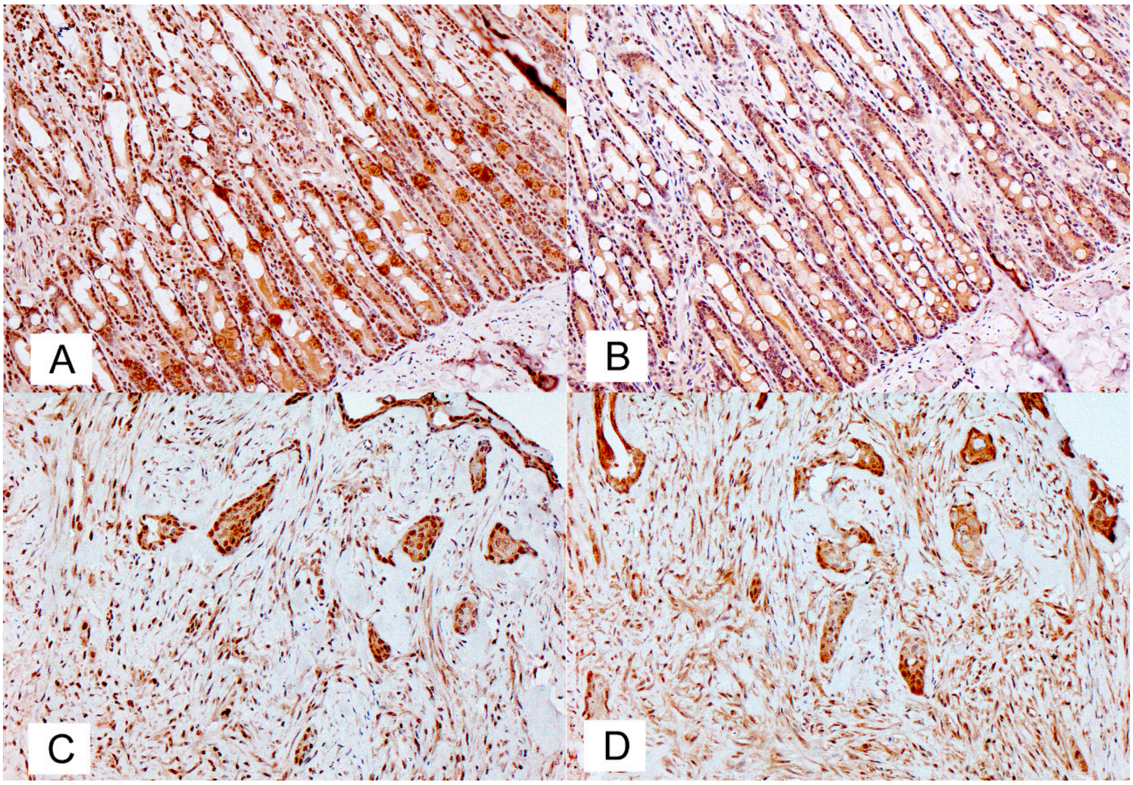

Figure S1. COX-1 positive and negative controls for immunohistochemistry. Feline small intestinal goblet cells served as positive control tissue. Photomicrographs of IHC staining results using rabbit anti-COX-1 antiserum (1:100) (S1A) and normal rabbit serum as a negative control (1:100, S1B). The chromogen is DAB (brown), and the counterstain is hematoxylin (blue). Replacement of the antiserum with normal serum revealed reduced goblet cell staining, but a high nonspecific background was present. Feline OSCC showed an intense COX-1 signal using the anti-serum (S1C), which was similar to the high nonspecific background signal from normal rabbit serum (S1D). COX-1 immunohistochemistry was not pursued further.
